# Direct reprogramming of adult somatic cells toward adventitious root formation in forest tree species: the effect of the juvenile–adult transition

**DOI:** 10.3389/fpls.2014.00310

**Published:** 2014-07-07

**Authors:** Carmen Díaz-Sala

**Affiliations:** Department of Life Sciences, University of AlcaláAlcalá de Henares, Spain

**Keywords:** age, auxin gradients, cell fate, developmental plasticity, epigenetics, gene expression, maturation, pluripotency

## Abstract

Cellular plasticity refers, among others, to the capability of differentiated cells to switch the differentiation process and acquire new fates. One way by which plant cell plasticity is manifested is through *de novo* regeneration of organs from somatic differentiated cells in an ectopic location. However, switching the developmental program of adult cells prior to organ regeneration is difficult in many plant species, especially in forest tree species. In these species, a decline in the capacity to regenerate shoots, roots, or embryos from somatic differentiated cells is associated with tree age and maturation. The decline in the ability to form adventitious roots from stem cuttings is one of the most dramatic effects of maturation, and has been the subject of investigations on the basic nature of the process. Cell fate switches, both in plants and animals, are characterized by remarkable changes in the pattern of gene expression, as cells switch from the characteristic expression pattern of a somatic cell to a new one directing a new developmental pathway. Therefore, determining the way by which cells reset their gene expression pattern is crucial to understand cellular plasticity. The presence of specific cellular signaling pathways or tissue-specific factors underlying the establishment, maintenance, and redirection of gene expression patterns in the tissues involved in adventitious root formation could be crucial for cell fate switch and for the control of age-dependent cellular plasticity.

## *DE NOVO* REGENERATION IN PLANTS

Plant tissues have extensive regenerative capacity, and entire plants can be developed from single cells or small cuttings (Xu and Huang, 2014). In plants, the possibility to regenerate roots, shoots, or embryos directly from cells other than root or shoot meristems, lateral root initials, or zygotes has been known for years and has been exploited in horticulture, agriculture, and forestry (Vasil, 2008). However, little is known about the mechanisms that enable a somatic differentiated cell to switch its fate into a pluripotent or totipotent cell that can develop a root, shoot or embryo, or repair damaged tissues.

Dedifferentiation, i.e., the loss of a specialized form or condition previously acquired during development that can be manifested by the loss of morphological cell identity or by the re-entry into the cell-cycle of non-dividing cells, has been a central concept in plant regeneration. Although apparent dedifferentiation and re-specification of cells seems to occur (Birnbaum and Sánchez Alvarado, 2008), whether acquisition of competence to regenerate organs occurs, as in animal cells, through dedifferentiation, via transdifferentiation or by the use of pre-existent totipotent or pluripotent cells in adult tissues remains unknown (Stocum and Zupane, 2008).

Recently, various publications on the mechanisms of *de novo* regeneration in non-recalcitrant model plant species demonstrate that there are many possible pathways and strategies for regeneration (Sena and Birnbaum, 2010). Callus formation has been considered a cell dedifferentiation process. However, Sugimoto et al. (2010) described that callus formation from specific *Arabidopsis* tissues involves a special population of starting pericycle-like cells distributed throughout the entire body of the plant. The process of callus formation is induced via a root developmental pathway, confirming that callus induction could not simply be the result of a dedifferentiation process. The mechanisms of induction and repression of callus formation are unknown. Several key regulators of auxin and cytokinin signaling pathways, as well as the involvement of intrinsic programs of cell development, and their interaction, function during callus formation (Chen et al., 2012; Ikeuchi et al., 2013).

If callus cells do not seem to have an undifferentiated state, and might have a root identity, the induction of shoots from callus through the application of cytokinins might involve the switch of one cell type into another, a process called transdifferentiation. In plant regeneration, transdifferentiation appears to be the process of regeneration in other cases, such as the plant root tip regeneration (Sena et al., 2009) or the direct origin of shoots from the hypocotyl and the root xylem pericycle cells, at sites normally involved in lateral root meristem formation, upon direct culture on a cytokinin-rich medium (Atta et al., 2009). The conversion of early lateral root meristem-like structures, induced upon direct culture in an auxin-rich medium, into shoots after treatments with cytokinins could be also considered a transdifferentiation process (Atta et al., 2009). These results demonstrate that xylem pericycle and pericycle-like cells, which are distributed throughout the entire body along the vasculature, could be pluripotent cells that directly originate the different morphogenic programs of callus, roots or shoots, depending on the stimulus in the culture medium. De Almeida et al. (2012) have described that pre-procambial cells can also act as niches for pluripotent and totipotent stem-like cells that are responsive to the auxin/cytokinin ratio resulting in *de novo* organogenic or embryogenic programs in the shoot apex of peach palm.

The molecular mechanisms underlying plant cell pluripotency of somatic differentiated cells are unknown. The capacity to recruit meristem or embryonic programs in response to a specific stimulus seems to be a key factor for switching cell fate into different developmental programs. Genes reported to have roles in maintaining stem cells or participating in embryonic programs, such as *WUSCHEL (WUS), WUSCHEL-RELATED HOMEOBOX (WOX), SHOOTMERISTEMLESS (STM)* or *LEAFY COTYLEDON (LEC)*, and the relevance of auxin and cytokinin signaling pathways in the regulation of key genes involved in the organization of stem cell niches have a role in *de novo* regeneration of *Arabidopsis thaliana, Brassica napus, Medicago truncatula, or Gossypium hirsutum* (Gordon et al., 2007; Chen et al., 2009; Kakani et al., 2009; Ledwon and Gaj, 2009; Su et al., 2009; Cheng et al., 2010; Elhiti et al., 2010; Elhiti and Stasolla, 2011; Yang et al., 2012; Feeney et al., 2013; Liu et al., 2014). In addition, other transcription factors with known function in meristem activity and stem cell maintenance, such as GIBBERELLIC-ACID INSENSITIVE, REPRESSOR of GAI and SCARECROW (GRAS) or WOUND INDUCEDDEDIFFERENTIATION1 (WIND) families were also related to cell reprogramming (Chupeau et al., 2013). Receptor-like kinase genes have been involved in the pluripotent condition leading to somatic embryogenesis, organogenesis and xylogenesis *in vitro* in *Cyclamen persicum* Mill (Savona et al., 2012). Many species of the genus *Kalanchoë* develop plantlets on the leaf margins. Asexual reproduction of these species involves genes related to embryonic development, such as *LEC*, in response to inductive signals in the leaves (Garces et al., 2007, 2014).

## RECALCITRANCE FOR *DE NOVO* REGENERATION IN FOREST TREE SPECIES. EFFECT OF AGE AND MATURATION

Recalcitrance for organogenesis or somatic embryogenesis is a major limitation in the clonal propagation of many woody species, especially forest tree species such as conifers. Endogenous and environmental factors such as genotype, tissue and timing of excision, phenology, tree maturation, light or temperature could be limiting for regeneration in these species (Bonga et al., 2010; Rutledge et al., 2013). Among them, tree maturation is one of the major limiting factors; it is well known that regeneration efficiency is much higher in tissues at earlier stages of development (George et al., 2008). Maturation is an age-related developmental process described in several plants which affects morphology and growth rate and is associated with the onset of flowering (Poethig, 2003). In trees, maturation has dramatic effects on a number of morphological and physiological traits, such as shoot height and diameter, foliar attributes, stomatal conductance, phostosynthesis, and respiration rates (Day et al., 2002; Day and Greenwood, 2011). Adventitious rooting is an essential but sometimes rate-limiting step in the clonal multiplication of elite tree germplasm, because the ability to form roots declines rapidly with age. Therefore, the decline in the ability to form adventitious roots from stem cuttings at maturation limits the success of vegetative propagation of adult individuals from tree species (Díaz-Sala et al., 1996; Day et al., 2002; Abarca and Díaz-Sala, 2009a). The threshold age at which this decrease occurs, and the rate of decline may vary among species and even among clones within species. In general, the bulk of evidence indicates that the ease in rooting of juvenile tissues of some trees is more a function of the ease in forming root initials than of the physical restriction for root emergence (Díaz-Sala et al., 1996; Goldfarb et al., 1998). This raises the question of whether trees maintain certain cells, which have not been determined to develop a specific organ, outside the meristematic region in a specific differentiated state that can easily access pluripotent or totipotent properties at a mature stage of development, and whether callus cells lose the differentiated characteristics of the tissue from which they originated as described for herbaceous plants (Sugimoto et al., 2010). Calli include a population of cells with several degrees of differentiation; and callus also displays several degrees of regeneration capacity. *In vitro* tissue culturists have experienced the diversity of callus morphology and their regeneration capacity. It is also known that callus cells from adult tissues of woody species maintain the committed state of the progenitor cells showing a lower proliferation rate and lower regeneration capacity than those induced from juvenile tissues (Hackett et al., 1990). In addition, for specific adventitious programs such as somatic embryogenesis, a highly genotype-dependent program, the induction of somatic embryos is difficult from tissues older than the zygotic embryo, often from the inmature state (Bonga et al., 2010). Therefore, the effect of the developmental transitions and the age of the tissues on the capacity of cells to be reprogrammed, and to induce a new developmental program in trees poses a major question. The loss of regeneration capacity associated with the juvenile–adult transition makes forest tree species representative and reliable systems to study how cell fate becomes fixed during development and how plant cells can manage to retain developmental plasticity.

## ADVENTITIOUS ROOT FORMATION IN FOREST TREE SPECIES

Adventitious root formation is a post-embryonic organogenic process in which roots are induced, in general, from determined, or differentiated cells that have not been specified to develop a root at positions where they do not normally occur during development. *De novo* induction of organs or whole organisms in an ectopic location, such as the appearance of roots from stem cuttings in plants, involve the induction of meristems from adult somatic cells that are not determined to originate a meristem in normal development. Adventitious root formation is usually induced in stem cuttings, which experience a stimulus, such as wounding, although it can also take place in intact plants in some species (Abarca and Díaz-Sala, 2009a). For most forest tree species, cambial cells or vascular parenchyma cells are the main progenitor cells to induce adventitious roots directly, i.e., without the induction of callus (Díaz-Sala et al., 1996; Ballester et al., 1999; De Klerk et al., 1999; Vidal et al., 2003; Rigal et al., 2012). These cells lose competence for *de novo* regeneration of roots with the age and maturation of the tree. Therefore, the different regeneration capacity of similar cell types at different developmental stages, and the absence of callus formation during the direct regeneration process, offers the opportunity to study both how cell fate becomes fixed during development, as well as a direct developmental switch, without passing through a developmentally non-identified callus cell.

While auxins are required to induce roots (Díaz-Sala et al., 1996; Goldfarb et al., 1998; Ballester et al., 1999; Fett-Neto et al., 2001), other factors also affect rooting (Abarca and Díaz-Sala, 2009a; Legué et al., 2014). The pronounced decline in the rooting potential of mature cuttings or mature tissue culture explants is paralleled by differences in the rooting response to auxin (Díaz-Sala et al., 1996; Greenwood et al., 2001). Although easy- and difficult-rooting lines from *Eucalyptus globulus* showed different levels of indole-3-acetic acid, auxins do not seem to be the limiting factor for the maturation-related decline to form adventitious roots in *Pinus, Castanea,* or *Quercus* species (Díaz-Sala et al., 1996; Ballester et al., 1999; Vidal et al., 2003). The initial hypothesis that non-competent mature tissues would not respond, or would respond more slowly, to auxin than competent tissues in terms of reorganization and cell division, was rejected in pine by Díaz-Sala et al. (1996) and Greenwood et al. (2001). Rooting-competent and non-competent cells respond similarly during the early stages of root induction; both types of cells re-enter the cell division cycle, but only cells at specific locations in juvenile tissues form an adventitious root meristem. This result added to the growing body of evidence that suggests that the loss of ability of mature tissues to form adventitious roots in response to auxin is not due to the lack of initial auxin responses (Díaz-Sala et al., 1996; Ballester et al., 1999; Greenwood et al., 2001). Therefore, in these cases, auxin seems to be a non-specific trigger of specific pre-set cell reaction patterns, and the lack of rooting ability could be a result of an intrinsic incapacity of cells to organize into a root meristem in response to auxin, perhaps due to the suppression of gene expression needed for cells to enter the root formation pathway during the earliest stages of adventitious root formation (Hutchison et al., 1999; Gil et al., 2003; Busov et al., 2004). The mechanisms underlying maturation-related decline of rooting ability, and other maturational changes, in forest tree species are unknown, but appear to be temporally regulated and synchronized by an array of signal transduction pathways that operate either independently or interacting with other signaling pathways, dependent on the development and very difficult to reverse (Day et al., 2002). According to Greenwood et al. (2001), rooting competence is ultimately a function of differential expression of genes affecting all phases of root meristem formation. Given that no specific auxin signal transduction pathways have been characterized in terms of rooting, elucidation of gene expression programs affecting rooting would be a worthwhile avenue of exploration.

## GENE REGULATION AND ADVENTITIOUS ROOT FORMATION IN FOREST TREE SPECIES

The cellular processes underlying adventitious root formation are complex, and involve cell reorganization, induction of cell divisions, the organization of a root primordium and root development and emergence (Abarca and Díaz-Sala, 2009a; Legué et al., 2014). However, the knowledge of the physiological and cellular changes involved in the rooting process does not necessarily lead to an understanding of the regulatory mechanisms involved. Reprogramming a differentiation pathway implies changes in cell division and patterns of cell differentiation requiring a change in the balance of the expression of hundreds of genes. The most promising approach to investigate the temporal distribution of specifically regulated transcripts in adventitious root development is the use of techniques directly detecting genes differentially expressed during different stages of the rooting process in different tissues (Abarca and Díaz-Sala, 2009a). In general, simple and synchronized experimental systems have been exploited for this purpose (Díaz-Sala et al., 1996; Hutchison et al., 1999; Lindroth et al., 2001; Brinker et al., 2004; Sánchez et al., 2007; Solé et al., 2008; Abu-Abied et al., 2012; Rigal et al., 2012). Different approaches have been followed to identify genes involved in adventitious root formation, from classical procedures of molecular biology (Goldfarb et al., 2003) to the sequencing of expression sequence tag (EST) collections enriched in cDNAs expressed in rooting-competent cuttings under inductive conditions, such as those obtained by differential displays of mRNAs (Díaz-Sala et al., 1997; Greenwood et al., 1997; Hutchison et al., 1999) or subtractive hybridization (Abarca and Díaz-Sala, 2009a). The sequencing of various tree genomes (Tuskan et al., 2006; Mackay et al., 2012; Petroli et al., 2012; Nystedt et al., 2013) has led to the development of high-throughput technologies used to follow the changes of expression of thousands of genes simultaneously. Global gene expression using microarray technology has been used to identify genes and pathways associated with adventitious rooting. Transcriptome analysis in stem cuttings revealed significant shifts during the very earliest stages of the process (Ramírez-Carvajal et al., 2009; Ramirez-Carvajal and Davis, 2010; Abu-Abied et al., 2012; Rigal et al., 2012), at the reorganization stage but before the onset of divisions leading to the formation of an adventitious root meristem (Sánchez et al., 2007; Solé et al., 2008; Vielba et al., 2011). In addition, the sequencing of tree genomes has also allowed the *in silico* identification and characterization of several multigene families.

### PLANT GROWTH REGULATORS AND OTHER SIGNALS

Plant growth regulators, mostly auxins, and other factors are crucial for root induction (Abarca and Díaz-Sala, 2009a; Negishi et al., 2011; Da Costa et al., 2013; Legué et al., 2014; Su and Zhang, 2014). Significant remodeling in gene networks involved in auxin-, gibberellin-, cytokinin- or ethylene-responsive genes, as well as genes that have been implicated in signaling such as the Ser/Thr protein kinases were detected during the initial stages in competent cuttings (Goldfarb et al., 2003; Ramirez-Carvajal and Davis, 2010; Rigal et al., 2012). Ramírez-Carvajal et al. (2009) have described that a cytokinin type-B response regulator, *PtRR13*, acts downstream of cytokinin to repress adventitious root formation in intact plants, and that reduced cytokinin signaling after shoot excision enables coordinated expression of ethylene, auxin, and vascularization pathways leading to adventitious root development in poplar.

*De novo* organ formation and cell specification are processes involving rearragements of tissue polarity, whereby the temporal and spatial distribution of auxin is a very important factor determining tissue polarization and patterning (Xu et al., 2006). Asymmetric auxin localization was detected in rooting-competent cambial tissues of Monterey pine after excision during the initial stages of the root induction process, before the onset of cell divisions, and at root meristem formation; however, no asymmetric auxin distribution was observed in the cambial cells of non-competent mature cuttings (Abarca et al., 2011; Brunoni et al., 2014). This result could indicate that rooting-competent tissues could retain a certain intrinsic capacity to accumulate auxin, perhaps by endogenous redistribution of PIN proteins (Sena et al., 2009). Auxin distribution largely depends on the dynamic expression and subcellular localization of the PIN auxin-carrier proteins (Friml, 2010). However, PIN activity can be modulated by other endogenous signals to trigger developmental decisions. Gibberellin treatment negatively affects the number of adventitious roots produced by wild-type poplar and hybrid aspen cuttings (Busov et al., 2006; Mauriat et al., 2014). Gibberellins appear to act by perturbing polar auxin transport, in particular auxin eﬄux in hybrid aspen, and both eﬄux and influx carriers in *Arabidopsis* (Mauriat et al., 2014). Alternatively, other signals, such as the stress originated by wounding (Grafi et al., 2011; Da Costa et al., 2013), could initiate regeneration by triggering cell fate or other local changes. No differences in the wounding stress response were observed between competent and non-competent cuttings (Greenwood et al., 1997). The level of mRNAs of genes that were not directly involved in root meristem organization, but were induced by other factors involved in the process such as the wounding-induced *PHENYLALANINE-AMMONIA-LYASE (PAL)*, was not dependent on the presence of exogenous auxin and was similar in competent and non-competent cuttings. These data suggest that the increased *PAL* level during the earliest stages of adventitious root formation does not seem to be a function of the rooting capacity of tissues, but rather a non-specific response to wounding. A higher transient increase of nitric oxide and a nitrate reductase gene expression were measured in juvenile cuttings of *E. grandis* (Abu-Abied et al., 2012) that may also affect auxin signaling. Regulatory functions via protein phosphorylation affecting proteins involved in rejuvenation of *Sequoia* and in regeneration have also been described (Chang et al., 2010; Wang et al., 2014).

### THE CONTINUUM OF CELL WALL, PLASMA MEMBRANE, CYTOSKELETON, AND CELL DIVISION

Gene expression analysis during the initial stages of adventitious root formation, at the cell reorganization stage, showed that genes coding for proteins involved in cell wall remodeling were the most highly expressed. *EXPANSIN* genes were induced in non-growing tissues before the resumption of cell division resulting in an adventitious root meristem in loblolly pine (Hutchison et al., 1999). Similarly, Rigal et al. (2012) demonstrated that the patterns of transcript abundance of several glycoside hydrolases, pectate lyases, pectin esterases, and expansins changed at this stage in poplar. Cytoskeleton genes, such as *ACTIN*, were upregulated in competent cutings during the initial stages of adventitious rooting in loblolly pine and *E. grandis* (Greenwood et al., 1997; Szwerdszarf et al., 2011). A protein with GTP-binding motifs, involved in regulating cell wall biosynthesis and actin organization, was associated with adventitious root formation and other processes requiring directional cell expansion, such as lateral root formation or the growth of root hairs, in poplar (Xu et al., 2012). In addition to the inductive effects of auxin, the effect of the RGD peptides is a necessary condition for rooting of hypocotyls from de-rooted adult plants of *Arabidopsis* (Díaz-Sala et al., 2002). These results provide support for the hypothesis that cell wall–plasma membrane–cytoskeleton interactions of specific cells could be involved in the rooting capacity of adult plants, perhaps by the stabilization of differentiation, cell division, and morphogenesis. Roycewicz and Malamy (2014) have suggested that intrinsic pathways that alter cell walls may be sufficient to promote lateral root formation by facilitating the separation of the cells overlying the lateral root primordium.The expression of the cell-cycle genes *CYCLIN-DEPENDENT KINASE (PtCDK*; Greenwood et al., 1997) and *PSTAIRE CDC2 (PcCDC2*; Lindroth et al., 2001) from pine suggests a role of *PcCDC2* in a premitotic stage and its expression could be an indicator of competence in which appropriate cells are capable of responding to auxin for adventitious rooting. Cell-cycle genes have been associated with cell competence and cell reprogramming during regeneration (Boucheron et al., 2002; Ishikawa et al., 2011). However, *PtCDK* does not seem to be related to the maturation-related loss of adventitious root formation. Perhaps re-entering to a formative cell division pathway in competent tissues, as opposed to proliferative cell division in non-competent tissues, requires other checkpoints.

### MERISTEM ACTIVITY AND CELL FATE DETERMINATION

Among the transcripts that show significant changes in expression at the reorganization stage of adventitious root formation, several transcription factors involved in embryonic programs and cell fate determination in meristems have been identified (Brinker et al., 2004; Sánchez et al., 2007; Ricci et al., 2008; Solé et al., 2008; Abarca and Díaz-Sala, 2009a,b; Vielba et al., 2011; Rigal et al., 2012; Trupiano et al., 2013; Brunoni et al., 2014). In poplar, the most highly modulated transcription factor group was the AP2/ERF family (Rigal et al., 2012). The AP2 subfamily is known to be involved in maintaining cells in a meristematic and/or division-competent state (Nole-Wilson et al., 2005). Among the 13 *ANT-LIKE* genes in poplar, several members of the *ANT-AIL* group were highly expressed at the initial stages of adventitious root formation, especially *PtAIL1*. Functional analysis clearly indicated that *PtAIL1* is a positive regulator of adventitious rooting that acts by promoting the formation of root primordia. Using activation tagging in poplar, Trupiano et al. (2013) have identified a gene encoding a transcription factor of the AP2/ERF family of unknown function (*PtaERF003*) that has a positive effect on both adventitious and lateral root proliferation. The function of *PtaERF003* is linked to the auxin signal transduction pathway, as suggested by the rapid induction and accentuated phenotypes of the transgenic plants in presence of the hormone.

In conifers, Brinker et al. (2004) investigated the gene expression pattern during adventitious root formation of lodgepole pine. Interestingly, a cell-fate meristem regulatory gene, *ZWILLE-LIKE*, was found during the earliest stages, suggesting a role in the initiation of the root development process. Similarly, Sánchez et al. (2007) and Solé et al. (2008) described a *SCARECROW-LIKE* gene in Monterey pine *(PrSCL1)* and in chestnut *(CsSCL1)*, and a *SHORT-ROOT* gene in Monterey pine *(PrSHR)* that may play a role during the earliest stages of adventitious root induction. In *Arabidopsis* and other plant species, such as rice or maize, the establishment of an embryonic root meristem involves members of the GRAS family of putative transcription factors which includes SCARECROW (SCR), SCARECROW-LIKE (SCL), and SHORT-ROOT (SHR) proteins (Helariutta et al., 2000; Sabatini et al., 2004). *PrSCL1, CsSCL1,* and *PrSHR* were predominantly expressed in roots, root primordia and in the cambial region of competent cuttings. Localized and transient increases of mRNA levels were observed in the cambial region and rooting-competent cells within the initial stages of the adventitious rooting process before the onset of cell divisions. The expression pattern of *PrSCL1* and *PrSHR* overlapped; *PrSCL1* was induced in the presence of the exogenous auxin needed for cuttings to root, whereas *PrSHR* induction was not dependent on exogenous auxin. These results suggest that auxin-dependent and auxin-independent pathways could be associated with the rooting capacity of tissues, and that *GRAS* genes may play a role during the earliest stages of adventitious rooting in pine (Sánchez et al., 2007; Solé et al., 2008). The *CsSCL1* signal was more diffuse and evenly distributed through the phloem and parenchyma in non-competent mature cuttings (Vielba et al., 2011). *GRAS* genes seem to also be involved in the rooting pathway associated with the enhancement of rooting by diphenylureas in Monterey pine (Ricci et al., 2008; Brunoni et al., 2014). The sequencing of conifer genomes (Mackay et al., 2012; Nystedt et al., 2013) has also led to the *in silico* identification and characterization of multigene families. Expression analysis of additional members of the pine GRAS family showed that the expression of specific members of the multigene family could be involved in complex transcript regulatory networks associated with tissue- and/or auxin-dependent pathways in root competent tissues (Abarca et al., 2011).

## COMPETENCE AND REPATTERNING: SEARCHING FOR INDETERMINANCY GENES

Unraveling the early events leading to the acquisition of cell pluripotency is a fundamental issue to understand cellular plasticity. There are two separate phenomena that appear to be basic steps in plant regeneration: (1) acquisition of competence to regenerate through dedifferentiation, transdifferentiation or by use of pre-existing totipotent cells; and (2) repatterning of regenerating tissues. Searching for master regulatory genes is essential in the future of adult cell fate switch studies, but searching for master rechanneling genes, which could be among the earliest events associated with fate switching by re-directing the differentiation state, is equally important (Abarca and Díaz-Sala, 2009b). In addition, the restriction of the reprogramming potential could be related to the presence of signals in the tissues that retain a physiological or developmental memory. Chromatin status and epigenetic mechanisms resulting in a specific nuclear architecture could be involved in the control of cellular plasticity (Bräutigam et al., 2013). Epigenetic regulation of vegetative phase change, dedifferentiation, and adventitious root formation has been described (Wang et al., 2011; Vining et al., 2013; You et al., 2014). In addition, epigenetically repressed embryonic programs could be presumably involved in callus repression and regeneration in post-embryonic tissues (Chen et al., 2012; Ikeuchi et al., 2013). The large-scale transcript profiles of plantlets and dedifferentiated protoplast-derived cells from *Arabidopsis* indicate that the epigenetic status of protoplast-derived cells differs from the well-established cultures, with protoplast-derived cells exhibiting changes in transposon reactivation and chromatin-associated genes (Chupeau et al., 2013). The genome sequencing of forest tree species and the development of transcriptomic tools, including next generation sequencing technologies, will allow a large-scale comparative analysis of transcriptomes of tissues with different degrees of determination and competence, facilitating the identification of new genes involved in cellular plasticity.

## Conflict of Interest Statement

The author declares that the research was conducted in the absence of any commercial or financial relationships that could be construed as a potential conflict of interest.
